# Intersection of Perceived COVID-19 Risk, Preparedness, and Preventive Health Behaviors: Latent Class Segmentation Analysis

**DOI:** 10.2196/50967

**Published:** 2023-10-24

**Authors:** Osaro Mgbere, Sorochi Iloanusi, Ismaeel Yunusa, Nchebe-Jah R Iloanusi, Shrey Gohil, Ekere James Essien

**Affiliations:** 1 Institute of Community Health University of Houston College of Pharmacy Houston, TX United States; 2 Department of Pharmaceutical Health Outcomes and Policy University of Houston College of Pharmacy Houston, TX United States; 3 Public Health Science and Surveillance Division Houston Health Department Houston, TX United States; 4 Department of Clinical Pharmacy and Outcomes Sciences University of South Carolina College of Pharmacy Columbia, SC United States; 5 Department of Internal Medicine, General Hospital Onitsha, Anambra State Nigeria

**Keywords:** COVID-19, latent class analysis, risk perception, preparedness, preventive health behaviors, Nigeria

## Abstract

**Background:**

COVID-19 risk perception is a factor that influences the pandemic spread. Understanding the potential behavioral responses to COVID-19, including preparedness and adoption of preventive measures, can inform interventions to curtail its spread.

**Objective:**

We assessed self-perceived and latent class analysis (LCA)–based risks of COVID-19 and their associations with preparedness, misconception, information gap, and preventive practices among residents of a densely populated city in Nigeria.

**Methods:**

We used data from a cross-sectional survey conducted among residents (N=140) of Onitsha, Nigeria, in March 2020, before the government-mandated lockdown. Using an iterative expectation-maximization algorithm, we applied LCA to systematically segment participants into the most likely distinct risk clusters. Furthermore, we used bivariate and multivariable logistic regression models to determine the associations among knowledge, attitude, preventive practice, perceived preparedness, misconception, COVID-19 information gap, and self-perceived and LCA-based COVID-19 risks.

**Results:**

Most participants (85/140, 60.7%) had good knowledge and did not perceive themselves as at risk of contracting COVID-19. Three-quarters of the participants (102/137, 74.6%; *P*<.001) experienced COVID-19–related information gaps, while 62.9% (88/140; *P*=.04) of the participants had some misconceptions about the disease. Conversely, most participants (93/140, 66.4%; *P*<.001) indicated that they were prepared for the COVID-19 pandemic. The majority of the participants (94/138, 68.1%; *P*<.001) self-perceived that they were not at risk of contracting COVID-19 compared to 31.9% (44/138) who professed to be at risk of contracting COVID-19. Using the LCA, we identified 3 distinct risk clusters (*P*<.001), namely, prudent or low-risk takers, skeptics or high-risk takers, and carefree or very high-risk takers with prevalence rates (probabilities of cluster membership that represent the prevalence rate [γ_c_]) of 47.5% (95% CI 40%-55%), 16.2% (95% CI 11.4%-20.9%), and 36.4% (95% CI 28.8%-43.9%), respectively. We recorded a significantly negative agreement between self-perceived risk and LCA-based segmentation of COVID-19 risk (κ=–0.218, SD 0.067; *P*=.01). Knowledge, attitude, and perceived need for COVID-19 information were significant predictors of COVID-19 preventive practices among the Onitsha city residents.

**Conclusions:**

The clustering patterns highlight the impact of modifiable risk behaviors on COVID-19 preventive practices, which can provide strong empirical support for health prevention policies. Consequently, clusters with individuals at high risk of contracting COVID-19 would benefit from multicomponent interventions delivered in diverse settings to improve the population-based response to the pandemic.

## Introduction

Since the declaration of COVID-19 as a public health emergency of international concern by the World Health Organization on January 30, 2020, the global case counts have reached 644 million, with more than 6.6 million deaths [1]. Despite recommended guidelines such as mask wearing, physical distancing, isolation, and good personal hygiene (handwashing) to prevent COVID-19 spread [2], some individuals tend to flout these guidelines, including government restrictions and rules. Government measures to contain the COVID-19 pandemic can only be effective with widespread compliance by the general public [3]. The fact that individuals in these challenging times act so differently indicates that the risk perception relating to this novel virus strongly differs between different places and individuals [4]. This situation, coupled with the pervasiveness of misinformation about the virus [5-9], is raising significant concerns, especially in Africa, where the fragile health systems put additional pressure on preparedness and effective pandemic response efforts. Consequently, COVID-19 risk perception may be a strong modifier of the evolution of the epidemic [4]. This means that access to and type of information received and political and economic situations may influence how people perceive the risk of COVID-19 and how they act on it, including their patterns of adhering to preventive actions [10].

In an early COVID-19 modeling study, Nigeria was identified as having a high coronavirus importation risk and high vulnerability, with moderate capacity to contain the outbreak [11]. The city of Onitsha in Nigeria is a highly populated town and home to the largest market in West Africa [12,13]. This city is known for the daily influx of people and frequent international, regional, and local interactions through commerce, which can facilitate the spread of COVID-19 within and outside the city. Previous studies in Onitsha have assessed COVID-19–related knowledge, attitude, and practice (KAP) [14]; public opinions regarding government response to the pandemic [3]; impact of COVID-19 misconceptions on the control efforts [15]; COVID-19–related information sources; and gaps [16]. However, it is unclear how the residents of Onitsha perceived the risk of COVID-19 and whether their initial risk perceptions informed their preparedness and decisions to adopt recommended protective measures. Risk perceptions tend to guide individuals’ judgments and evaluations of threats and can limit public compliance with and response to information from public health authorities [4,10,17,18].

Cori et al [4] espouse the need to apply established theories of risk perception research to COVID-19 and use this knowledge to improve health risk communication, build trust, and contribute to collaborating governance. People who perceive greater risks are believed to be more motivated to implement protective behaviors [10,17,18]. Although the global consequences of COVID-19 are hard to predict, an assessment of the initial population-level response to the disease in Onitsha regarding risk perception, preparedness, and preventive health behaviors could provide helpful information for interventions and improve current and future public health response. Understanding the risk perceptions of community residents is critical for planning, risk communication, and intervention.

In light of the foregoing situation, this study aims to (1) assess the association of self-perceived risk of COVID-19, preparedness, and preventive practices among residents of Onitsha, (2) apply latent class analysis (LCA) to systematically segment the heterogeneous sample population into the most likely distinct risk clusters by using selected measures, and (3) examine the predictive factors for COVID-19 preventive practices among Onitsha city residents. An understanding and application of the interplay of these factors in the real world could influence behavior change, improve risk management decision-making, and inform a more targeted and effective COVID-19 intervention strategy to prevent and control the disease spread in the city.

## Methods

### Data Source, Study Design, and Participants

We conducted a secondary analysis of cross-sectional data obtained from a KAP survey in Onitsha, Anambra, Nigeria, in March 2020 (period of the pandemic before the government-mandated lockdown on March 29, 2020) [14]. A convenience sampling method was used to recruit 140 study participants from different representative locations within the city of Onitsha that includes commercial markets and housing units. The city of Onitsha is the largest commercial city in south-eastern Nigeria and has a population of nearly 8.1 million residents with a population density of 4100 per square kilometer [19]. This survey was conducted through in-person interviews of consenting adults aged 18 years and older living and working in Onitsha. A more detailed description of the survey instrument used, data collection procedures, and the study area can be found in the study by Iloanusi et al [14].

### Analytical Measures

The analytical measures used in this study were obtained from baseline data collection [14]. The data set captured KAP data as quantitative and categorical measures coded dichotomously as poor or good [14]. The quantitative part represented the KAP indices. COVID-19 misconceptions were assessed based on the study of Iloanusi et al [14] and categorized as none, low, and high [15]. Participants were asked to indicate what they considered to be their risk perception for COVID-19 by using a 5-point Likert scale (ranging from not at risk to extremely at risk). This was classified into 3 categories (not at risk at all, somewhat or likely at risk, likely or extremely likely at risk) and, subsequently, reclassified as 2 categories (not at risk vs at risk). To assess the participant’s perceived level of preparedness, they were asked how prepared they were for the impending COVID-19 outbreak, with the response options being “undecided or not prepared at all,” “somewhat prepared for COVID-19,” and “prepared for COVID-19.” This was later classified into 2 categories for subsequent analysis, with the last 2 options grouped as one and referred to as “prepared for COVID-19.” To assess information gaps, participants were asked if they needed COVID-19 information and coded dichotomously as Yes or No [14,17]. The demographic characteristics of the sample population used in this study have been described in detail previously by Iloanusi et al [14].

### Statistical Analysis

Using the chi-square test, we conducted univariable analyses of measures of interest, namely, risk perception for COVID-19 outbreaks, KAP, participants’ level of preparedness, misconceptions, and the COVID-19 information gap. To examine the relationships between KAP indices by self-perceived COVID-19 risk, preparedness, and information (gap), we conducted a linear regression analysis with a density contour overlay to show the potential patterns (clusters) within the measures. The bivariate fit models produced equations that described the relationships between the measures.

LCA was used to fit a latent class model to determine the most likely cluster or latent class for each observable measure by using an iterative expectation-maximization algorithm [20,21]. LCA was considered appropriate for understanding and exploring the meaning behind the participants’ risk perception. Using LCA allowed for estimating the population characteristics derived from the sample data, adjusting the measurement error, and determining the number of classes [22]. The LCA produced the latent class prevalence (γ_c_) and the conditional probabilities (ρ) for each cluster and response category. Estimates of the effect size and likelihood ratio logworth obtained from a contingency table analysis of expected counts for cluster membership by levels or categories of a Y column were used to quantify differences within the response scales. The final LCA model fitness was determined using negative log-likelihood (–log-likelihood), Bayesian information criterion, and Akaike information criterion to compare clusters with the smallest values of each, indicating the best fit. In addition, we considered entropy values, latent class probabilities, and interpretability of the model class identified in selecting the final model [21,23]. Based on these characteristics, definitions for each latent class were created. We identified 3 distinct risk clusters named as “prudent or low-risk takers,” “skeptics or high-risk takers,” and “carefree or very high-risk takers.” To enhance the ease of interpretation and clearer application to practice, the risk clusters were recoded dichotomously as “not at risk (0)” for prudent or low-risk takers and “at risk (1)” for both skeptics or high-risk takers and carefree or very high-risk takers. Furthermore, we determined the independent association between self-perceived COVID-19 risk and LCA-assessed COVID-19 risk and the study population characteristics by using a chi-square test or Fisher exact test (cell number <5), when applicable.

To identify factors associated with the adoption of COVID-19 preventive practice, we conducted multivariable logistic regression analyses to estimate the unadjusted odds ratios and adjusted odds ratios (aORs) along with 95% CIs and the corresponding *P* values for each factor. We applied the mosaic plot data visualization with the associated chi-square test to display the intersection between self-perceived COVID-19 risk, LCA-based risk subgroup, and preventive practices by perceived preparedness. All statistical tests performed were 2-tailed, with a probability value of .05 used as the minimum threshold for declaring statistical significance. Data management, statistical analyses, and visualizations were conducted using SAS JMP Statistical Discovery Software (version 16.2; SAS Institute). This study was reported following the STROBE (Strengthening The Reporting of Observational Studies in Epidemiology) statement [24] and aligns with the minimum specific reporting requirements in the cross-sectional study checklist [25].

### Ethics Approval

All relevant ethical guidelines, including institutional review board approval and oral informed consent, were provided by all participants and documented during the primary data collection period [14]. Data used for this study were codified and anonymized to protect confidentiality and ensure individual participants' privacy. The study protocol for this secondary data analysis was reviewed and approved (approval 00002363) by the institutional review board of the University of Houston.

## Results

### Descriptive Analysis of Measures

The descriptive characteristics of the study participants have been presented in detail by Iloanusi et al [14]. The demographic characteristics of this study population is displayed in Multimedia Appendix 1. The univariable analysis of the measures evaluated is shown in Table 1. We noted a significant difference in participants’ COVID-19 knowledge levels, with more than half of them (85/140, 60.7%; *P*=.01) having a high knowledge of the disease. However, the majority (85/140, 60.7%; *P*<.001) did not perceive they were at risk of contracting COVID-19. About 62.9% (88/140; *P*=.047) of the participants had some misconceptions about COVID-19, while only 37.1% (52/140; *P*=.047) had no misconceptions about the disease. As much as 74.6% (102/137; *P*<.001) indicated the need for more COVID-19–related information. However, most participants (93/140, 66.4%; *P*<.001) indicated that they were prepared for the COVID-19 pandemic. Overall, the study participants (N=140) were indifferent (*P*>.05) in their attitude and preventive practice levels.

**Table 1:** Univariable analysis of the study measures (N=140).

Characteristic		Values, n (%)	*P* value	
**Knowledge**				.01^a^
	Low	55 (39.3)		
	High	85 (60.7)		
**Attitude**				.13^b^
	Poor	61 (43.6)		
	Good	79 (56.4)		
**Preventive practice**				.39^b^
	Poor	65 (46.4)		
	Good	75 (53.6)		
**COVID-19 misconception**				.047^c^
	None	52 (37.1)		
	Low	55 (39.3)		
	High	33 (23.6)		
**Perceived need for COVID-19 information**				<.001^d^
	No	35 (25.5)		
	Yes	102 (74.5)		
**Perceived preparedness**				<.001^d^
	Not prepared for COVID-19	47 (33.6)		
	Prepared for COVID-19	93 (66.4)		

^a^Significant at *P*<.01.

^b^Not significant (*P*>.05).

^c^Significant at *P*<.05.

^d^Significant at *P*<.001.

### Relationships Between KAP Indices

**Figure 1:**
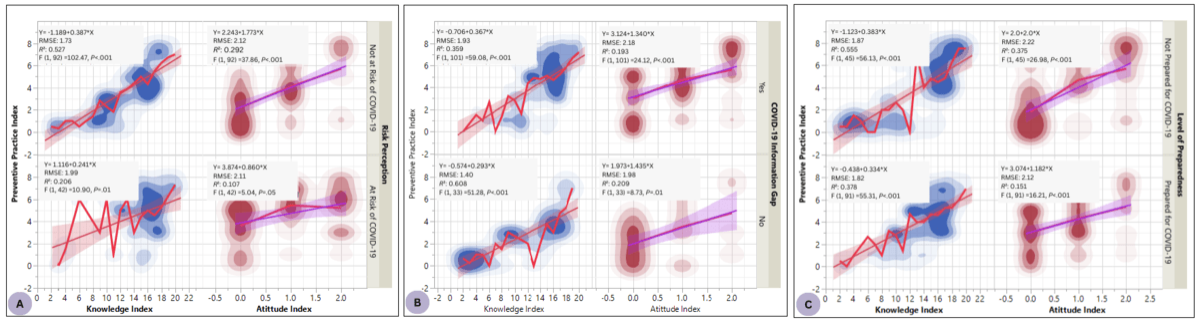
(A) Relationships among knowledge, attitude, and preventive practice indices by self-perceived COVID-19 risk. (B) Relationships among knowledge, attitude, and preventive practice indices by perceived COVID-19 information gap. (C) Relationships among knowledge, attitude, and preventive practice indices by perceived COVID-19 pandemic preparedness.

The relationships among KAP indices by COVID-19 risk perception are shown in Figure 1A. A linear relationship was observed across the measures among individuals who perceived that they were at risk or not at risk of COVID-19. The contours show the regions of data density relative to the indices. Among the at-risk group, increased COVID-19 knowledge index and attitude index scores resulted in an increased level of preventive practice implementation (*R*^2^=0.206 for knowledge index; *P*<.001 vs *R*^2^=0.107 for attitude index; *P*=.05). In contrast, knowledge index and attitude index significantly (*P*<.001) predicted as much as 52.7% and 29.2% of the preventive practice levels implemented by study participants who perceived they were not at risk of contracting COVID-19.

Figure 1B depicts the relationships among KAP indices by perceived COVID-19 information gap with the bands indicating the concentration of participants relative to their scores. Within participants who perceived that they had gaps in COVID-19–related information, increased knowledge index and attitude index resulted in a significant linear increase in their adoption of recommended preventive practices, as indicated by the preventive practice indices (*R*^2^=0.359 vs *R*^2^=0.193, respectively). However, participants who did not perceive any gap in COVID-19 information had higher predictive associations among KAP indices. For instance, a positive increase in knowledge index and attitude index resulted in a corresponding increase in preventive practice indices and coefficients of determination of 60.8% (knowledge index, *P*<.001) and 20.9% (attitude index, *P*<.01).

The relationships among KAP indices by perceived preparedness with the contours indicating the density of participants relative to their index values are shown in Figure 1C. Within participants who perceived that they were prepared for the COVID-19 pandemic, increased knowledge index and attitude index brought about a significant (*P*<.001) linear increase in their adoption of recommended preventive practices, as reflected in the preventive practice index values (*R*^2^=0.378 vs *R*^2^=0.151, respectively). However, participants who perceived themselves unprepared for the COVID-19 pandemic recorded comparatively higher predictive values. For instance, a positive increase in knowledge index and attitude index resulted in a corresponding increase in preventive practice index and coefficients of determination of 55.5% (knowledge index, *P*<.001) and 37.5% (attitude index, *P*<.001).

### LCA Model

The LCA model parameter estimates with the conditional probabilities (ρ) for each cluster and response category are presented in Table 2. Following the study population heterogeneity concerning a set of manifest variables, we used LCA to identify 3 significantly (*P*<.001) distinct most likely clusters or latent classes (homogeneous subgroups or segments) of individuals. Those were named after their unique characteristics as prudent or low-risk takers (γ=47.5%), skeptics or high-risk takers (γ=16.2%), and carefree or very high-risk takers (γ=36.4%). The key characteristics of the prudent or low-risk takers include high COVID-19 knowledge (ρ=95.2%), good attitude (ρ=85.4%), self-perception of not being at risk of COVID-19 (ρ=58.9%), perceived preparedness for COVID-19 pandemic (ρ=62%), experienced information gap (ρ=81.5%), no misconceptions (ρ=66.5%), and good preventive practices (ρ=87.5%). Among individuals characterized as skeptics or high-risk takers*,* 59.9% of them had high knowledge of COVID-19, a poor attitude (ρ=66.6%), claimed not to be at risk of COVID-19 (ρ=54.9%), perceived themselves as being prepared for the disease (ρ=87%), had very serious information gaps (ρ=98.5%), many misconceptions (ρ=97.9%), and surprisingly, practiced good prevention strategies against COVID-19 *(*ρ=82.5%). However, carefree or very high-risk takers had low knowledge (ρ=81.3%), poor attitude (ρ=72.7%), self-perception of not being at risk of COVID-19 (ρ=84.5%), perceived preparedness for COVID-19 pandemic (ρ=60%), experienced information gap (ρ=53.5%), had moderate misconceptions (ρ=68.1%), and implemented poor preventive practices (ρ=99.4%).

**Table 2:** Parameter estimates from latent class segmentation analysis.^a^

Parameter, category		n	Probability (ρ) of latent class membership (%)				Effect size		LRL^b^
			Prudent or low-risk takers	Skeptics or high-risk takers	Carefree or very high-risk takers				
Overall (γ_c_)		140	47.5	16.2	36.4	—^c^		—	
**Prevention practice**							0.83^d^		24.80
	Poor	65	12.5	17.5	99.4				
	Good	75	87.5	82.5	0.6				
**Knowledge**							0.71^d^		17.16
	Low	55	4.8	40.1	81.3				
	High	85	95.2	59.9	18.7				
**Attitude**							0.57^e^		10.20
	Poor	61	14.6	66.6	72.7				
	Good	79	85.4	33.4	27.3				
**Risk perception**							0.28^f^		2.39
	Not at risk of COVID-19	94	58.9	54.9	84.5				
	At risk of COVID-19	44	41.1	45.1	15.5				
**Perceived preparedness**							0.20^f^		1.36
	Not prepared for COVID-19	47	38	13	40				
	Prepared for COVID-19	91	62	87	60				
**COVID-19 information gap**							0.38^f^		4.81
	Yes	101	81.5	98.5	53.5				
	No	35	18.5	1.5	46.5				
**Misconception**							0.48^e^		8.15
	No	52	66.5	2.1	31.9				
	Yes	88	33.5	97.9	68.1				

^a^The overall probabilities of cluster membership (γ_c_) and the conditional probabilities (ρ) for each cluster are shown for each response category. Model fit statistics best fit estimates: negative log-likelihood=563.04; Bayesian information criterion=1239.07; and Akaike information criterion=1172.08.

^b^LRL: likelihood ratio logworth. A logworth value above 2 corresponds to significance at the .01 significance level (*P*<.01).

^c^Not applicable.

^d^Large effect size.

^e^Medium effect size.

^f^Small effect size.

### Associations Between Self-Perceived and LCA-Based Segmentation of COVID-19 Risks and Selected Measures

Table 3 shows the associations among KAP, misconception, perceived information needs, preparedness, COVID-19 risk perception, and LCA COVID-19 risk assessment. Most participants (94/138, 68.1%; *P*<.001) self-perceived that they were not at risk of COVID-19 compared to 31.9% (44/138; *P*<.001) who professed to be at risk of contracting COVID-19. Based on the LCA assessment, we recorded no statistical difference (*P*>.05) between the 2 groups (67/136, 49.3% vs 69/136, 50.7%). However, with the LCA classification, we recorded statistically significant variations (*P*=.05) across all measures considered, except perceived preparedness compared to self-perceived risk assessment, where knowledge (*P*=.01) and preventive practice (*P*=.03) were the only significant sources of variations. Based on the participant’s characteristics, the LCA generally tends to classify more participants as at risk of contracting COVID-19 than the self-perceived assessment. For instance, the proportion of participants who self-perceived themselves as being at risk were 5, 3, 3.8, and 3 times more than those who had poor COVID-19 knowledge, attitude, preventive practice, and high misconception, respectively, based on the LCA-based assessment of their COVID-19 risk status.

**Table 3:** Associations between self-perceived and latent class analysis–based segmentation of COVID-19 risk and selected measures.

Measure		Self-perceived COVID-19 risk (n=138)				Latent class analysis–based COVID-19 risk assessment (n=136)					
		Not at risk, n (%)	At risk, n (%)	*P* value		Not at risk, n (%)		At risk, n (%)		*P* value	
Overall		94 (68.1)	44 (31.9)	<.001^a^		67 (49.3)		69 (50.7)		.86^b^	
**Knowledge**					.01^c^						<.001^a^
	Low	43 (31.2)	10 (7.3)			2 (1.5)		50 (36.8)			
	High	51 (37)	34 (24.6)			65 (47.8)		19 (13.9)			
**Attitude**					.68^b^						<.001^a^
	Poor	42 (30.4)	18 (13)			7 (5.2)		53 (38.9)			
	Good	52 (37.7)	26 (18.8)			60 (44.1)		16 (11.8)			
**Preventive practice**					.03^d^						<.001^a^
	Poor	49 (35.5)	14 (10.1)			9 (6.6)		52 (38.2)			
	Good	45 (32.6)	30 (21.7)			58 (42.7)		17 (12.5)			
**COVID-19 misconception**					.96^b^						<.001^a^
	None	34 (24.6)	17 (12.3)			37 (27.2)		13 (9.6)			
	Low	37 (26.8)	17 (12.3)			27 (19.8)		26 (19.1)			
	High	23 (16.7)	10 (7.3)			3 (2.2)		30 (22.1)			
**Perceived need for COVID-19 information**					.33^b^						.045^d^
	No	26 (19.1)	9 (6.6)			54 (40)		46 (34.1)			
	Yes	66 (48.5)	35 (25.7)			12 (8.9)		23 (17)			
**Perceived preparedness**					.44^b^						.59^b^
	Not prepared for COVID-19	34 (24.6)	13 (9.4)			25 (18.4)		22 (16.2)			
	Prepared for COVID-19	60 (43.5)	31 (22.5)			42 (30.8)		47 (34.6)			

^a^Significant at *P*<.001.

^b^Not significant (*P*>.05).

^c^Significant at *P*<.01.

^d^Significant at *P*<.05.

### Multivariable Logistic Regression Model

The multivariable logistic regression model showing the unadjusted and aORs of the measures and COVID-19 preventive practice is presented in Table 4. Of all the measures included in our model, knowledge, attitude, and perceived need for COVID-19 information were the participants’ only significant predictors of COVID-19 preventive practices (entropy *R*^2^=0.3352). Participants who had high COVID-19 knowledge and good attitude toward the management of COVID-19 were 11 (aOR 11.22, 95% CI 4.34-28.97; *P*<.001) and 3 (aOR 2.93, 95% CI 1.14-7.55; *P*=.03) times more likely to have good COVID-19 preventative practices, respectively. Surprisingly, participants who needed more COVID-19 information were about 4 times more likely (aOR 3.92, 95% CI 1.36-11.30; *P*<.01) to have good COVID-19 preventive practices compared to those who experienced no information gap.

**Table 4:** Multivariable logistic regression model of COVID-19 preventive practice.^a^

Measures		Unadjusted			Adjusted			
		Odds ratio (95% CI)	*P* value		Odds ratio (95% CI)		*P* value	
**Knowledge**				<.001^b^				<.001^b^
	Low (reference)	1			1			
	High	14.63 (6.26-34.18)			11.22 (4.34-28.97)			
**Attitude**				<.001^b^				.03^c^
	Poor (reference)	1			1			
	Good	4.70 (2.29-9.63)			2.93 (1.14-7.55)			
**Risk perception for COVID-19**				.03^c^				.41^d^
	Not at risk at all (reference)	1			1			
	At risk	2.33 (1.10-4.95)			1.51 (0.57-3.99)			
**COVID-19 misconception**				.14^d^				.99^d^
	Low (reference)	1			1			
	High	0.60 (0.85-3.27)			0.99 (0.37-2.65)			
**Perceived need for COVID-19 information**				.01^b^				.01^e^
	No (reference)	1			1			
	Yes	4.21 (1.82-9.71)			3.92 (1.36-11.30)			
**Perceived preparedness**				.67^d^				.85^d^
	Not prepared for COVID-19 (reference)	1			1			
	Prepared for COVID-19	1.16 (0.58-2.35)			1.10 (0.41-2.92)			

^a^Model statistics: McFadden’s pseudo R-square (*R*^2^ [U])=0.34; Akaike information criterion=139.25; Bayesian information criterion=158.76. Normal approximation used for ratio confidence limits effects. Tests and confidence intervals of odds ratios are Wald test–based.

^b^Significant at *P*<.001.

^c^Significant at *P*<.05.

^d^Not significant (*P*>.05).

^e^Significant at *P*<.01.

### Intersection of Perceived COVID-19 Risk, Latent Class Risk Subgroup, Preparedness, and Preventive Practice

**Figure 2:**
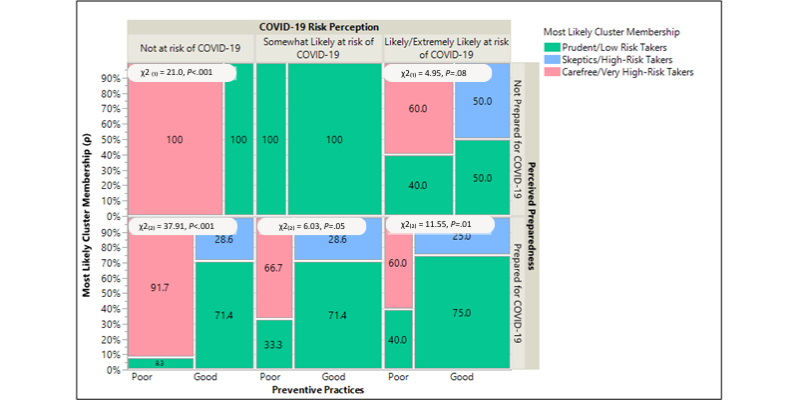
Mosaic plot of the associations between self-perceived COVID-19 risk and latent class analysis–based risk assessment of most likely cluster membership by perceived preparedness.

The mosaic plot in Figure 2 displays the associations between self-perceived COVID-19 risk, LCA-based risk subgroup, and preventive practices by perceived preparedness. Among participants who perceived themselves as not at risk of COVID-19 but were prepared, 71% (5/7) were characterized as prudent low-risk takers and adopted good preventive strategies (*P*<.001). Similarly, most of the participants who perceived themselves as somewhat likely or likely or extremely likely at risk of contracting COVID-19 and were prepared for the pandemic ended up as prudent low-risk takers (5/7, 71%; *P*=.05 vs 12/16, 75%; *P*=.01, respectively) and implemented good preventive measures. However, most participants, including those who were prepared and not prepared for the COVID-19 pandemic, practiced the recommended preventive measures poorly (60%-100%) and were classified as carefree or very high-risk takers based on the LCA model. Only 29% (2/7; *P*=.05) and 25% (4/16; *P*=.01) of the participants who implemented good preventive practices were skeptics or high-risk takers and self-perceived themselves as likely at risk and extremely likely at risk of contracting COVID-19 (Figure 2). Overall, we recorded a significant negative agreement between self-perceived and LCA-based segmentation of the risk groups (κ=–0.2182, SD 0.0665; *P*=.01).

## Discussion

### Principal Findings

During the initial days of the COVID-19 pandemic, the Nigerian government, like many other governments, implemented a series of preventive practices that included public lockdown, handwashing, use of hand sanitizer, wearing of face masks, and social distancing in public places, to curtail the spread of COVID-19. Although these interventions are effective, they require voluntary behavior change and compliance by citizens, who found this challenging to achieve and monitor. Our study investigates the role of behavioral determinants on self-perceived and LCA-based COVID-19 risks and the adoption of COVID-19 preventive practices. Our findings revealed that adopting good COVID-19 preventive practices among residents of Onitsha was generally driven by increased knowledge and improved attitude toward COVID-19 infection moderated by their risk perception status, information gaps, and level of preparedness. Several COVID-19–related studies have previously established the relationships between COVID-19 knowledge, attitude, and prevention practices [14,26,27]. Individuals who believed they were at low risk of developing COVID-19 were more likely to engage in unhealthy or risky behaviors [28].

Using the LCA, we identified 3 distinct risk clusters (*P*<.001), namely, prudent or low-risk takers, skeptics or high-risk takers, and carefree or very high-risk takers, with prevalence rates of 47.5%, 16.2%, and 36.4%, respectively. Individuals who were high-risk to very high–risk takers tended to have many misconceptions, experienced COVID-19 information gaps, self-perceived themselves as not being at risk of contracting COVID-19, and therefore adopted poor preventive practices. A previous study has highlighted the existence of fundamental misconceptions that hindered compliance with prevention practices among Onitsha residents [15]. Similarly, COVID-19 information gaps and low diffusion due to government delays in initiating awareness campaigns using accessible and efficient channels of information have been associated with poor COVID-19 preventive practices [16,29,30].

Accurate public risk perceptions are critical to effectively managing COVID-19, especially considering that people’s behavior can fundamentally influence and alter the spread of a pandemic [31-33]. In an attempt to assess “COVID-19 risk as feelings” (self-perceived) and “COVID-19 risk as analysis” (LCA-based assessment), we recorded a significant negative agreement between self-perceived risk and LCA-based segmentation of COVID-19 risk (κ=–0.218, SD 0.067; *P*<.01). Consequently, participants with poor knowledge, attitude, preventive practice, and high misconception who claimed not to be at risk of COVID-19 infection were reported to be 3-5 times more at risk of contracting COVID-19 when assessed using LCA. These measures also had significant effect sizes in determining the probability of latent class membership. Although risk perceptions influence individual protective behaviors [17,34], our findings indicate that an individual’s perception of risk may not necessarily correlate positively with the actual analyzed risk. A pessimistic bias, that is, perceptions of risk that are (much) higher than the actual risk, is more likely for new risks such as COVID-19 that are perceived as uncontrollable [35].

LCA was considered appropriate for understanding and exploring the meaning behind risk perceptions in our study population. Threat appraisal and risk perception are essential determinants of the public’s willingness to cooperate and adopt health-protective behaviors during pandemics, including frequent handwashing, physical distancing, avoiding public places, and wearing face masks [36-38]. These risk perceptions guide individuals’ judgments and evaluations of threats and can limit public compliance with and response to information that authorities communicate [4,10,17,18,39]. LCA has been used to study various issues in vulnerable populations, including mental health among Black youth [40], young Malawian adults with or at risk for HIV [41], and adolescent perceptions of in-school discrimination [42].

Of all the measures included in our multivariable model, only knowledge, attitude, and perceived need for COVID-19 information were significant predictors of COVID-19 preventive practices among the participants. Previous studies have also documented the associations between the KAP indices in this population [14]. However, it was surprising that participants who experienced the COVID-19 information gap were about 4 times more likely to have good preventative practices against the disease. This suggests that public health messages by the Nigeria Center for Disease Control and other governmental agencies may have been responsible for a positive behavioral change toward risk aversion during the early stage of the COVID-19 pandemic [3,16]. Residents of Onitsha, like many others worldwide, were faced with a new and unfamiliar health threat that could result in deaths, coupled with the fact that information on the disease was initially limited and changed more often with time. Fear of the unknown has been associated with the absence of information and, when encountered in sufficiently predictable and controllable contexts, could facilitate positive responses [43,44]. This may have been the case in our study population.

The intersection of self-perceived COVID-19 risk, LCA most likely risk clusters, perceived preparedness, and preventive practice indicates that most participants who self-perceived themselves as not being at risk of COVID-19 and had poor preventive practices (carefree ones) were noted to be at a very high risk of contracting COVID-19 infection when assessed using LCA. This make-belief may have brought about complacency on the public part, enforced by false or misleading COVID-19 narratives promoted by some groups to discredit legitimate public health measures [45]. The advent of social media and web-based platforms, which provide a fertile medium for disinformation to flourish, has been widely acknowledged as a threat to global efforts toward ending the pandemic [15,16,40]. This situation raises important concerns, especially in Africa, where the fragile health systems put additional pressure on preparedness and effective pandemic response efforts. However, our study noted that all the participants who claimed nonpreparedness but the likelihood of being somewhat at risk of COVID-19 accurately matched the LCA-based assessment classification as prudent or low-risk takers. People who perceive greater risks are believed to be more motivated to implement protective behaviors [10,17,18]. Efforts to improve pandemic preparedness and response to the next pandemic might benefit from greater investment in risk communication and community engagement and in developing strategies to counter misinformation and boost individuals' confidence in public health guidance [15,16,39,44-46]. Since latent class membership helps explain the patterns of individuals’ scores on the indicator variables used to derive the classes, the LCA solutions therefore represent typologies that can help researchers and practitioners understand commonalities and differences across individuals, which have implications for both practice and future research [21].

### Strengths and Limitations

This study’s findings should be interpreted with caution because of some limitations. This study is a secondary data analysis based on a cross-sectional study conducted during the early onset of the COVID-19 pandemic using a nonprobability convenience sampling method with a small sample size. Therefore, the data may be subject to sampling and potential response biases due to social desirability and unobserved confounding, leading to nonrepresentativeness of the population and possibly overestimating the direction and strength of associations. Similarly, the use of LCA is limited by the fact that individuals are assigned to classes based on the probability of likely cluster membership predicated on the scores of the indicator variables [21]. As a result, the exact number or percentage of class memberships cannot be guaranteed due to some level of misclassifications. Further, the complexity of the classes may inadvertently engage in a naming fallacy, wherein the class name does not accurately reflect the class membership [21]. Therefore, from the foregoing situation, definite causality cannot be inferred, and the generalizability of our findings is limited. The government intervention’s role in messaging may also have impacted an individual’s risk assessment and response to preventive care practices [3]. Finally, it is essential to note that the availability and uptake of the COVID-19 vaccines more than 2 years after the data used in this study were collected may have decreased self-perceived risk and adherence to COVID-19 preventive measures over time [47].

Despite these limitations, our study’s strength lies in applying latent class segmentation analysis to reveal important insights into the relationships between behavioral measures and COVID-19 infection risk. Although self-report may not allow for the assessment of actual behavior due to social desirability, the application of LCA allowed for culturally competent and context-specific risk classification, which may be particularly useful in identifying subgroups of individuals who could benefit from a common intervention based on their shared characteristics [21,23]. Data used for this study were collected during the early phase of the COVID-19 pandemic and, thus, provide rich baseline information that could be used by public health authorities to assess COVID-19 response efforts or for current and future pandemic intervention planning.

### Conclusion

The LCA clustering patterns highlight the impact of modifiable risk behaviors on COVID-19 preventive practices, which can provide strong empirical support that may encourage behavior change, especially during the COVID-19 pandemic or any future outbreak of similar infectious diseases. Consequently, clusters with individuals at high risk of contracting COVID-19 would benefit from multicomponent interventions delivered in diverse settings to improve the population response to the COVID-19 pandemic. This finding may also offer clinicians the opportunity to refer their patients at high risk of contracting the disease to social workers or psychologists for behavioral counseling. In addition, understanding the role of risk perceptions in motivating people to engage in preventive behavior by public health authorities may also help with intervention program planning and designing evidence-based risk communication strategies.

## Appendix

Multimedia Appendix 1 Demographic characteristics of the study population.

## Abbreviations

aOR: adjusted odds ratio
KAP: knowledge, attitude, and practice
LCA: latent class analysis
STROBE: Strengthening The Reporting of Observational studies in Epidemiology

## Data Availability

Data for this study can be made available upon reasonable request from the corresponding author.
