# Supplementary figures and images for: Intersection of Perceived COVID-19 Risk, Preparedness, and Preventive Health Behaviors: Latent Class Segmentation Analysis
Source: Online J Public Health Inform. 2023 Oct 24;15:e50967. doi: 10.2196/50967 (PMC10689050; doi:10.2196/50967)

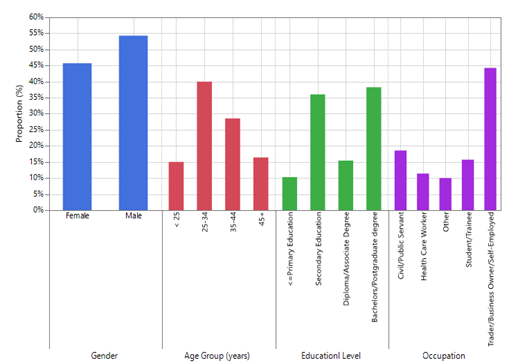

Supplement: Multimedia Appendix 1 [file ojphi_v15i1e50967_app1.png]
